# Sex differences in acute telestroke care: more to the story

**DOI:** 10.3389/fneur.2023.1203502

**Published:** 2023-06-21

**Authors:** Theresa Sevilis, Amanda Avila, Mark McDonald, Mariecken Fowler, Renata Chalfin, Murtaza Amir, Gregory Heath, Mohammed Zaman, Lorianne Avino, Caitlyn Boyd, Lan Gao, Thomas Devlin

**Affiliations:** ^1^TeleSpecialists, LLC, Fort Myers, FL, United States; ^2^Public Health, University of Tennessee at Chattanooga, Chattanooga, TN, United States; ^3^Mathematics, University of Tennessee at Chattanooga, Chattanooga, TN, United States; ^4^Neurology Department, University of Tennessee Health Science Center, Memphis, TN, United States

**Keywords:** telestroke, healthcare disparities, stroke, women, acute stroke care, thrombolytics

## Abstract

**Background:**

Previous studies have shown sex differences in stroke care. Female patients have both lower thrombolytic treatment rates with OR reported as low as 0.57 and worse outcomes. With updated standards of care and improved access to care through telestroke, there is potential to reduce or alleviate these disparities.

**Methods:**

Acute stroke consultations seen by TeleSpecialists, LLC physicians in the emergency department in 203 facilities (23 states) from January 1, 2021 to April 30, 2021 were extracted from the Telecare by TeleSpecialists^™^ database. The encounters were reviewed for demographics, stroke time metrics, thrombolytics candidate, premorbid modified Rankin Score, NIHSS score, stroke risk factors, antithrombotic use, admitting diagnosis of suspected stroke, and reason not treated with thrombolytic. The treatment rates, door to needle (DTN) times, stroke metric times, and variables of treatment were compared for females and males.

**Results:**

There were 18,783 (10,073 female and 8,710 male) total patients included. Of the total, 6.9% of females received thrombolytics compared to 7.9% of males (OR 0.86, 95% CI 0.75–0.97, *p* = 0.006). Median DTN times were shorter for males than females (38 vs. 41 min, *p* < 0.001). Male patients were more likely to have an admitting diagnosis of suspected stroke, *p* < 0.001. Analysis by age showed the only decade with significant difference in thrombolytics treatment rate was 50–59 with increased treatment of males, *p* = 0.047. When multivariant logistic regression analysis was performed with stroke risk factors, NIHSS score, age, and admitting diagnosis of suspected stroke, the adjusted odds ratio for females was 0.9 (95% CI 0.8, 1.01), *p* = 0.064.

**Conclusion:**

While treatment differences between sexes existed in the data and were apparent in univariate analysis, no significant difference was seen in multivariate analysis once stroke risk factors, age, NIHSS score and admitting diagnosis were taken into consideration in the telestroke setting. Differences in rates of thrombolysis between sexes may therefore be reflective of differences in risk factors and symptomatology rather than a healthcare disparity.

## Introduction

Sex differences have been reported in acute stroke care and outcomes over the last few decades (1, 2). Analysis of this issue has focused on the difference in risk factors, acute care received, and post stroke care including secondary stroke prevention. The issue has proven to be complex partially due to the variance in female stroke risk factors throughout a lifetime associated with hormonal stages. Females on average have a longer lifespan which contributes to an increased cumulative lifetime risk of stroke. Many risk factors are shared by both males and females but affect the sexes differently.

There are multiple studies highlighting sex differences in acute stroke treatment including thrombolytic treatment rates and door to needle (DTN) times. One study from a German stroke registry with over 53,000 patients had an adjusted OR of 0.87 (95% CI 0.78–0.96) (3). Foerch et al.’s analysis included thrombolytics treatment rate with both intravenous and intra-arterial thrombolysis. The multivariate analysis adjusted for age, pre-stroke disability, clinical symptoms, vascular risk factors and type of stroke. In addition to the lower treatment rates, Asdaghi et al. (4), reports females are less likely to receive thrombolysis with DTN times of <60 min unadjusted OR 0.83, (95% CI 0.71–0.97, *p* = 0.02). The median time for males in this study was 69 min (IQR 52-91) and females was 73 min (IQR 55-97), *p* = 0.005. The lower thrombolytics utilization in females may contribute significantly to the worse outcomes seen in females, in addition to their older age at onset, pre-stroke functional status, and decreased access to rehabilitation (2, 5).

One of the barriers to treatment identified in a study from Denmark including 5,356 stroke events was a delay in symptoms onset to hospital arrival time (median delay of 20 min) for females compared to males (6). Various socioeconomic factors are suspected to contribute to the differences in treatment and outcome, including living alone, ability to articulate symptoms, and healthcare literacy (7). While studies show a narrowing in the treatment gap between sexes over the last decade (8), further identification of the barriers to acute stroke treatment for females is necessary to help improve this gap.

Over recent decades, telestroke has been improving access to acute stroke care around the world. One study suggests that telestroke services may reduce health care disparities as there was no difference seen in the treatment rates between males and females in their telestroke network (9). More robust evidence supporting a reduction of healthcare disparities within telestroke networks, would further support the ongoing implementation of telestroke networks. We sought to leverage the large TeleCare by TeleSpecialists^™^ database to further characterize possible disparities in acute stroke treatment between sexes within a telemedicine care model.

## Methods

Acute stroke consultations seen by TeleSpecialists, LLC physicians in the emergency department in 203 facilities (23 states) from January 1, 2021 to April 30, 2021 were extracted from the Telecare by TeleSpecialists^™^ database. The TeleCare by TeleSpecialists database is comprised of prospectively collected data. The encounters were reviewed for age; sex; date seen; last known normal (LKN); arrival time; consult call time; needle time; thrombolytics candidate; premorbid modified Rankin Score (p-mRS); National Institute of Health Stroke Scale (NIHSS) score; screen time; stroke risk factors including hypertension, diabetes mellitus, hyperlipidemia, atrial fibrillation, coronary artery disease and previous stroke; antithrombotic use; admitting diagnosis of suspected stroke; and reason not treated with thrombolytic. Admitting diagnosis of suspected stroke was determined based on the diagnosis code of the neurologist for consultation. If a code for ischemic stroke or a symptom code consistent with stroke were utilized, then the case was included as a suspected stroke. Diagnosis codes that were clear alternatives to an ischemic stroke were excluded. The reasons not treated were classified as subjective vs. objective reasons. Objective reasons included last known normal >4.5 h, use of DOACs within 48 h, coagulopathy, thrombocytopenia, intracranial intra-axial neoplasm, current or previous ICH, GI bleeding within 21 days, stroke in last 3 months, recent major surgery, other diagnosis suspected, and other. Subjective reasons included resolved symptoms, no focal deficits, patient declined, and no disabling symptoms.

The treatment rates, Door-to-needle (DTN) times, p-mRS scores, NIHSS scores, screen times, arrival to notification times, pre-notification by EMS, and LKN to arrival times were compared for females and males.

Additional analyses were performed to evaluate the causes of variation in treatment. Patients were age stratified by decade to assess variations in treatment based on age. Multivariate logistic regression analysis was performed using age, median NIHSS scores, stroke risk factors, admitting diagnosis of suspected stroke, and thrombolytics treatment. The reasons not treated were compared between sexes based on subjective and objective categories. Subgroup analysis of patients with an admitting diagnosis of suspected stroke was also performed for all variables.

The demographic and clinical characteristics of male and female patients were compared using appropriate statistical analyses. Continuous variables were presented as mean ± standard deviation for normally distributed data and as median with interquartile range (IQR) for non-normally distributed data. Categorical variables were reported as frequency with percentage. To assess differences in normally distributed continuous variables, the Student’s *t*-test was used, while the Mann–Whitney U test was employed for non-normally distributed variables. The Chi-squared test was utilized to examine the association between categorical variables. Variables with a *p-*value < 0.1 in the uni-factor regression analysis, as well as potential variables related to outcomes, underwent further validation through multi-factorial regression analysis. A multi-factor logistic regression was conducted to investigate the difference in thrombolytic treatment rate between males and females, taking into account potential confounding factors such as age, NIHSS score, stroke risk factors, and stroke status. The results of the logistic regression model, including adjusted odds ratios (OR), 95% confidence intervals (CI), and *p*-values, were reported. A *p*-value of < 0.05 was considered statistically significant. All statistical analyses were performed using R version 4.1.1.

## Results

The total number of acute stroke consultations extracted was 18,783 (10,073 female and 8,710 male). Of the 10,073 female patients seen, 691 (6.86%) received thrombolytics compared to 689 (7.91%) of the 8,710 of males seen (OR 0.86, 95% CI 0.75–0.97, *p* = 0.006). Male patients were significantly more likely than female patients to have each individual stroke risk factor and antithrombotic use at presentation (Table 1).

**Table 1:** Characteristics and stroke risk factors for females vs. males.

	All patients (*N* = 18,783)	Female (*N* = 10,073)	Male (*N* = 8,710)	*p*-value
**Age (years)**	66.0 ± 16.3	65.9 ± 17.2	66.1 ± 15.1	0.3934
Stroke risk factors				
Hypertension	8,633 (46%)	4,453 (44.2%)	4,180 (48%)	**<0.001**
Diabetes mellitus	3,560 (19%)	1,785 (17.7%)	1,775 (20.4%)	**<0.001**
Hyperlipidemia	4,996 (26.6%)	2,512 (24.9%)	2,484 (28.5%)	**<0.001**
Atrial fibrillation	1,589 (8.5%)	780 (7.7%)	809 (9.3%)	**<0.001**
Coronary artery disease	1,922 (10.2%)	745 (7.4%)	1,177 (13.5%)	**<0.001**
Prior stroke	2,934 (15.6%)	1,498 (14.9%)	1,436 (16.5%)	0.007
Prior anticoagulant	1,872 (10%)	920 (9.1%)	952 (10.9%)	**<0.001**
Prior antiplatelet	4,693 (25%)	2,348 (23.3%)	2,345 (26.9%)	**<0.001**

Data presented as *N* (%) except for age which is mean (SD). Bold values indicate a significance at *p* < 0.05.

**Figure 1:**
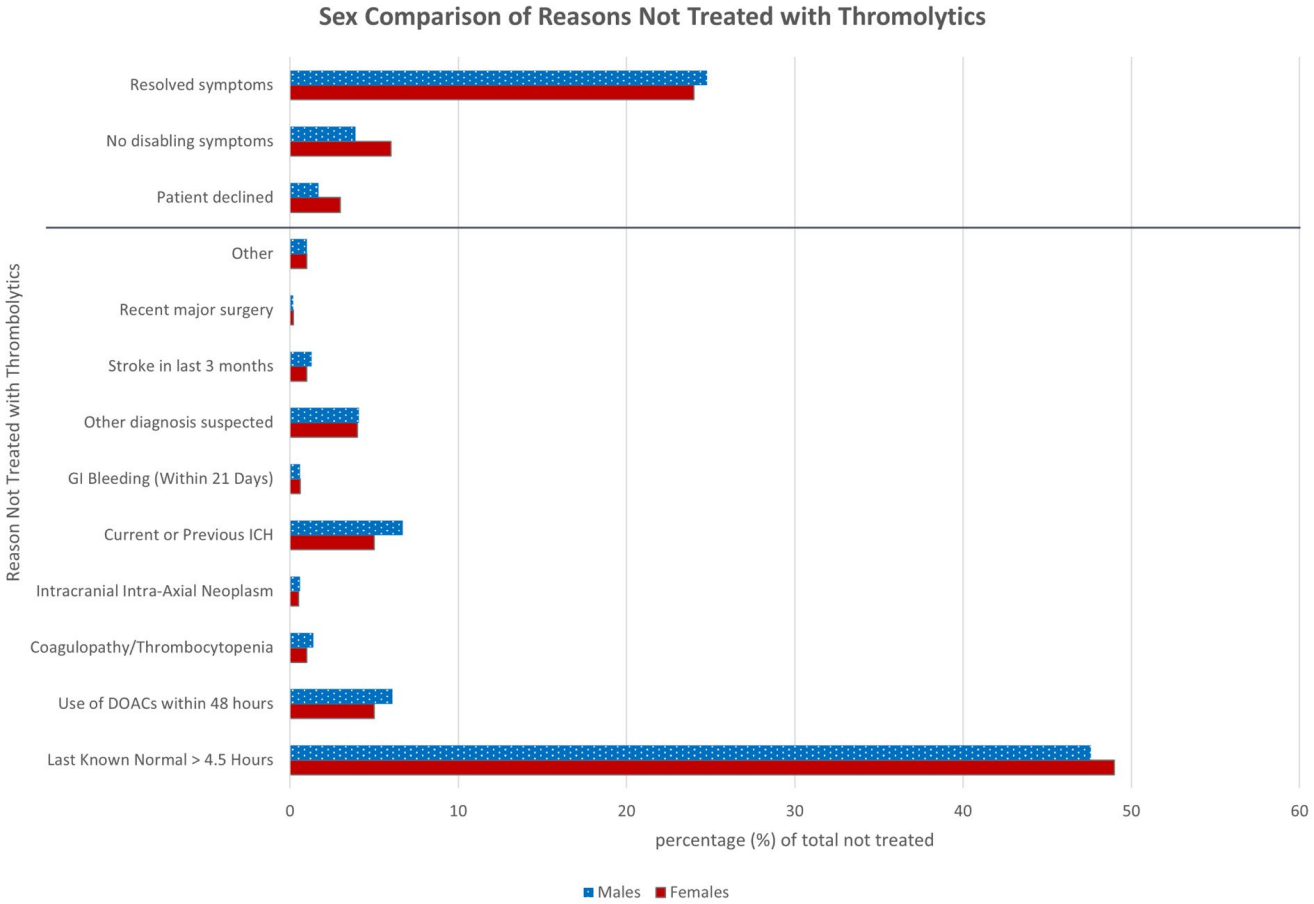
Sex comparison of reasons not treated with thrombolytics. Reasons patients were not treated with thrombolytics in the acute window of 4.5 h compared between sexes. There were no patients excluded from the 8,021 males not treated and 1 female excluded from the 9,382 females not treated due to unavailable data. The objective reasons are below the gray line and the subjective reasons are to the above of the gray line. DOACs, Direct oral anticoagulants; ICH, intracranial hemorrhage; GI, gastrointestinal.

When comparing female and male patients treated with thrombolytics, the median DTN times were significantly shorter for males (38 min) compared to females (41 min), *p* < 0.001. Male patients were more likely to present via EMS (male 57.4% and female 54.6%), and female patients more likely to present via triage (male 41.7% and female 44.4%) (Table 2). Male patients were also more likely to have an EMS prenotification (male 22% and female 20.2%). There was not a significant difference in the percentage of female patients (49%) presenting within the 4.5-h thrombolytics window compared to males (49.7%), *p* = 0.372. The median arrival to notification of neurologist times were longer for female patients at 10.7 vs. 10 min, *p* = 0.002 while there was no difference in the LKN to arrival between sexes (Table 2). There was no difference in NIHSS scores, p-mRS, or physician screen times. More male patients had an admitting diagnosis of suspected stroke then female patients (Table 2). Female patients had 11% greater odds of declining thrombolytics or having non-disabling symptoms as the reason for not being treated with thrombolytics. Figure 1 details the breakdown of reasons not treated by sex.

**Table 2:** Univariate analysis of stroke metric times and features of initial encounter.

	Female (*N* = 10,073)	Male (*N* = 8,710)	*p*-value
Treated with thrombolytics	691 (6.9%)	689 (7.9%)	**0.007**
Median DTN time (min)	38 (31, 55)	41 (29, 51)	**<0.001**
EMS presentation	5,498 (54.6%)	5,000 (57.4%)	**<0.001**
Neurologist pre-notification	1,962 (20.2%)	1,848 (22.0%)	**0.003**
Median arrival to notification (min)	10.7 (5, 22)	10 (5, 21)	**0.002**
Last known normal to arrival (min)	173 (64, 612)	174 (64, 579)	0.688
Presenting within 4.5 of symptom onset	4,918 (48.8%)	4,314 (49.5%)	0.372
Median NIHSS	2 (0, 6)	2 (0, 6)	0.860
Median p-mRS	0 (0, 3)	0 (0, 3)	0.535
Physician screen time (min)	20 (14, 27)	20 (14, 27)	0.117
Admitting diagnosis of suspected stroke	4,803 (47.7%)	4,529 (52.0%)	**<0.001**
**Reason not treated with thrombolytics**			
Subjective contraindication	3,067 (30.5%)	2,440 (28.0%)	**<0.001**
Objective contraindication	7,006 (69.5%)	6,270 (72.0%)	**0.005**

Data presented as *N* (%) except for median DTN, median arrival to notification, last known normal to arrival, median NIHSS, median p-mRS which is median (IQR). Bold Values indicate a significance at *p* < 0.05.

DTN, Door to Needle; min, Minutes; NIHSS, National Institute of Health Stroke Scale; p-mRS, Premorbid Modified Rankin Scale.

**Figure 2:**
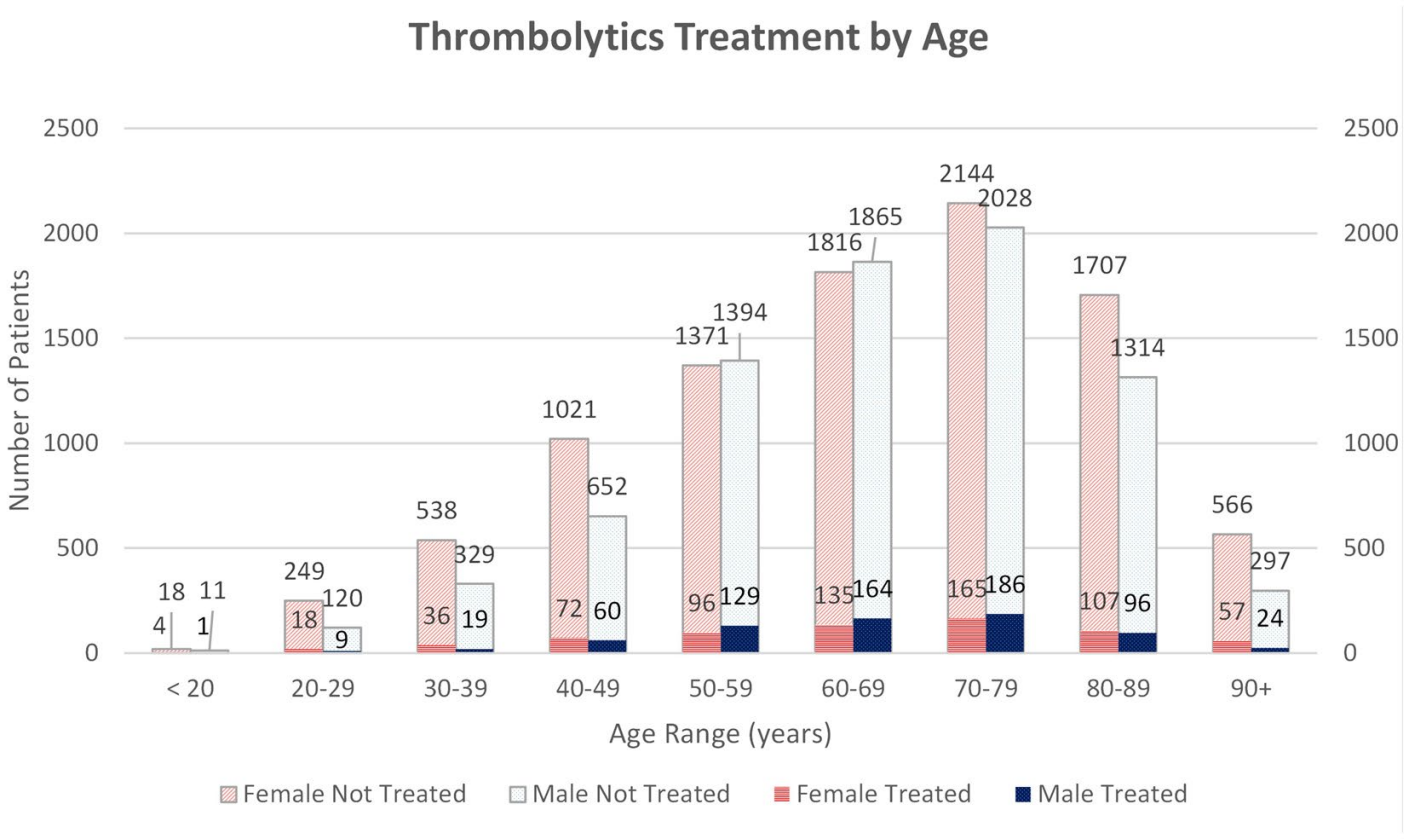
Thrombolytic treatment rates of each sex are represented by age. 10,056 female patients included and 8,698 male patients. 17 females and 12 males were excluded due to age not available. Patients are divided into decades with an initial group of <20 and last ≥90. Number of untreated and treated with thrombolytics patients of each sex represented in each decade.

Further analysis of age was performed by grouping by decades, with 19 and younger patients and 90 and older patients as the initial and final groups, respectively. In this analysis, the only statistically significant difference seen was in the age group 50–59 years, with males more likely than females to receive thrombolytics, *p* = 0.046 (Figure 2). While there was not a significant difference between sexes, there was a high treatment rate of both females <20 and ≥90, 18.2 and 9.1%, respectively.

A multivariate logistic regression analysis was performed for difference in thrombolytics treatment rates between sexes with males as the reference including potentially confounding variables of age, NIHSS, stroke risk factors, and admitting diagnosis of suspected stroke (Table 3). The OR for females was 0.9 (CI 0.8, 1.01, *p* = 0.064). The variables with significant differences were age with OR 0.99 (CI 0.99, 0.99, *p* < 0.001), median NIHSS score OR 1.08 (CI 1.07, 1.09, *p* < 0.001), history of atrial fibrillation OR 0.39 (CI 0.3, 0.5, *p* < 0.001), previous stroke OR 0.64 (0.54, 0.77, *p* < 0.001), and admitting diagnosis of suspected stroke 14.46 (CI 11.98, 17.6, *p* < 0.001).

**Table 3:** Multivariate logistic regression model of thrombolytics between female and male adjusting for confounding variables.

	Adjusted OR	95% CI	*p*-value
Female	0.9	[0.8, 1.01]	0.064
Male	Ref	Ref	Ref
Age, mean (SD)	0.99	[0.99. 0.99]	**<0.001**
NIHSS, median (IQR)	1.08	[1.07, 1.09]	**<0.001**
Hypertension	0.97	[0.85, 1.12]	0.705
Diabetes mellitus	0.94	[0.8, 1.1]	0.437
Hyperlipidemia	1.03	[0.88, 1.21]	0.678
Atrial fibrillation	0.39	[0.3, 0.5]	**<0.001**
Coronary artery disease	1.01	[0.83, 1.23]	0.9141
Prior stroke	0.64	[0.54, 0.77]	**<0.001**
Admitting diagnosis of suspected stroke	14.46	[11.98, 17.6]	**<0.001**

The adjusted ORs were calculated using males as the reference group. SD, Standard Deviation; IQR, Interquartile Range; ED, Emergency Department. Bold values indicate a significance at *p* < 0.05.

The variable that stood out in the multivariate logistic regression analysis was the admitting diagnosis of suspected stroke so the subgroup analysis of patients with an admitting diagnosis of suspected stroke was performed (Table 4). Within this subgroup analysis differences in age, some of the prior stroke risk factors, median DTN times persisted, but the difference in thrombolytics treatment rates was no longer present.

**Table 4:** Subgroup comparison of female and male patients with suspected stroke admitting diagnosis.

	Female patients (*N* = 4,803)	Male patients (*N* = 4,529)	*p*-value
Age, mean (SD)	68.3 ± 16.3	67.1 ± 14.3	**<0.001**
History of hypertension, *N*(%)	2,445 (50.9%)	2,425 (53.5%)	**0.023**
History of diabetes mellitus, *N* (%)	972 (20.2%)	1,050 (23.2%)	**0.003**
History of atrial fibrillation, *N*(%)	476 (9.9%)	509 (11.2%)	0.079
History of coronary artery disease, *N*(%)	424 (8.8%)	699 (15/4%)	**<0.001**
Prior stroke, *N*(%)	885 (18.4%)	867 (19.1%)	0.462
Prior anticoagulation, *N*(%)	515 (10.7%)	576 (12.7%)	0.374
Prior antiplatelet, *N*(%)	1,328 (27.6%)	1,405 (31.0%)	0.495
Treated with thrombolytics, *N*(%)	628 (13.1%)	628 (13.9%)	0.276
Median DTN time (min) median (IQR)	41.0 [30.0, 54.0]	38.0 [29.0, 49.0]	**0.002**
EMS presentation, *N*(%)	2,787 (58.0%)	2,626 (58.0%)	0.885
Neurologist pre-notification, *N*(%)	958 (19.9%)	1,040 (23.0%)	**<0.001**
Presenting within 4.5 h of symptom onset, *N*(%)	2,452 (59.5%)	2,328 (59.8%)	0.781
NIHSS, median (IQR)	2 (1.0, 6.0)	2 (1.0, 6.0)	0.208
p-mRS, median (IQR)	0 (0.0, 3.0)	0 (0.0, 3.0)	**0.007**
Physician screen time (min), median (IQR)	21.0 (15.0, 30.0)	21.0 (15.0, 30.0)	0.942
Arrival to notification (min), median (IQR)	10 (5.0, 21.0)	10 (5.0, 19.0)	0.050
Last known normal to arrival (min), median (IQR)	173.0 (63.0, 606.0)	167.0 (62.0, 590.0)	0.501
**Reason not treated with thrombolytics**			
Subjective contraindication, *N*(%)	1,361 (28.3%)	1,218 (26.9%)	0.222
Objective contraindication, *N*(%)	3,442 (71.7%)	3,311 (73.1%)	0.222

Patients with a diagnosis code other than ischemic stroke or symptoms that could potentially be an ischemic stroke were excluded from the analysis, creating a group of patients in which the evaluating neurologist was considering stroke as a potential diagnosis. SD, standard deviation; IQR, interquartile range. Bold Values indicate a significance at *p* < 0.05.

## Limitations

The limiting factors in this study were the lack of final diagnosis of stroke and the 90 day modified Rankin scores. Assessing the outcomes in addition to the treatment rates to assure that the difference correlated with both 90 day and long-term outcomes would help solidify the argument for reduction of disparities in care between sexes.

## Discussion

While univariate analysis of thrombolytics treatment between sexes showed a difference consistent with recent studies with an OR 0.86 (95% CI 0.75–0.97, *p* = 0.006), these differences were no longer significant in the multivariate logistic regression analysis (OR 0.9 95% CI 0.8, 1.01, *p* = 0.064). This suggests that there is not a true difference in the treatment rates of strokes but rather a difference in presenting symptoms that trigger an acute stroke assessment between sexes. The treatment rates in our study did not use a denominator of stroke diagnosis but rather a denominator of stroke alerts called in the emergency department. Evidence shows that females present with more non-specific symptoms which may lead to a broader net being cast when calling a stroke alert on female patients (7). This is suspected to have contributed to the higher number of female patients in the study and the lower percentage with an admitting diagnosis of suspected stroke. While the chief complaints were not available in this study, the significant difference in subjective reasons as contraindications in primary analysis which includes non-disabling symptoms supports this. While in the subgroup analysis of admitting diagnosis of suspected stroke, there is no difference in subjective vs. objective contraindications.

In univariate analysis, males were found to have a significantly increased likelihood of having each of the stroke risks factors collected. This would imply that the male population is at higher risk of stroke from a younger age so despite the increased longevity of females there may be a longer duration high-risk time period for males. This combined with the admitting diagnosis of suspected stroke were the main driving forces in the lack of significance in the multivariate analysis of thrombolytics treatment rates. Subgroup analysis showed increased stroke risk factors in males as well but not as significantly or for all variables.

There was a three-minute difference in the door to needle times for male and female patients in both primary and subgroup analysis which is likely explained by difference in presentation. Male patients were more likely to be transported by EMS thereby expediting the evaluation process, especially when combined with pre-notification allowing the neurologist to be on screen at the time of the patient’s arrival. A limiting factor of screen time is that it does not mean direct patient interaction time. That was not a measured metric so females may have had longer direct encounter time prior to decision making if more males had pre-notification. Female patients were also more likely to have subjective reasons for not being treated with thrombolytics in the primary analysis which could lead to longer discussions. Although this was not supported with the data for median screen time being equal at 20 min. While statistically significant, it is unlikely that the three-minute difference in treatment time translates into a clinically significant difference in stroke outcomes.

Prior studies have reported a delay in female patients presenting as a cause for differences in treatment rates. Neither the primary or subgroup analysis support this as there was no significant difference in the percentage of patients presenting within 4.5 h and the median last known normal to arrival times.

Our study supports the prior findings that patients receiving acute stroke treatment via telestroke do not appear to have significant differences in thrombolytics rates based on sex. Both the multivariate and subgroup analysis of admitting diagnosis of suspected stroke support this. Further studies to assess for improvement in outcomes in female patients treated via telestroke are warranted. It would also be of interest to assess if the availability of teleneurology follow up impacts the outcomes of strokes for female patients as this plays a significant role in patient outcomes.

## Data availability statement

The raw data supporting the conclusions of this article will be made available by the authors, without undue reservation.

## Ethics statement

Ethical review and approval was not required for the study on human participants in accordance with the local legislation and institutional requirements. Written informed consent from the patients/participants or patients/participants legal guardian/next of kin was not required to participate in this study in accordance with the national legislation and the institutional requirements.

## Author contributions

TS, AA, MZ, and TD: substantial contribution to conception and design. TS, MM, MF, RC, MA, MZ, and LA: acquisition of data. TS, AA, MM, MZ, GH, LG, and TD: analysis and interpretation of data. TS: drafted the manuscript. All authors revised the manuscript and gave final approval of the version to be published.

## Funding

Financial support for this study was provided by the Neuroscience Innovation Foundation.

## Conflict of interest

TS, AA, MM, MF, RC, MA, MZ, LA, CB, and TD are employed by TeleSpecialists, LLC.

The remaining authors declare that the research was conducted in the absence of any commercial or financial relationships that could be construed as a potential conflict of interest.

## Publisher’s note

All claims expressed in this article are solely those of the authors and do not necessarily represent those of their affiliated organizations, or those of the publisher, the editors and the reviewers. Any product that may be evaluated in this article, or claim that may be made by its manufacturer, is not guaranteed or endorsed by the publisher.
